# Intention to shift from short-acting to long-acting methods of contraceptives and its associated factors in Gondar city, northwest Ethiopia: Using the theory of planned behavior

**DOI:** 10.3389/frph.2022.882916

**Published:** 2022-09-13

**Authors:** Edom Retta Fekadu, Telake Azale, Resom Berhe, Adane Nigusie, Zelalem Nigussie Azene, Maereg Wolde

**Affiliations:** ^1^School of Pharmacy, College of Medicine and Health Sciences, University of Gondar, Gondar, Ethiopia; ^2^Department of Health Education and Behavioral Sciences, College of Medicine and Health Sciences, University of Gondar, Gondar, Ethiopia; ^3^Department of Women's and Family Health, School of Midwifery, College of Medicine and Health Sciences, University of Gondar, Gondar, Ethiopia

**Keywords:** intention, Ethiopia, contraception [^*^methods], theory of planned behavior (TPB), Gondar

## Abstract

**Background:**

Despite its effectiveness, the intention, and utilization of long-acting contraceptive methods are very low in many developing countries, and the use of long-acting contraceptive methods has not kept pace with that of short-acting methods such as oral contraceptives and injectables. Therefore, this study aimed to assess the intention of using long-acting contraceptive methods and associated factors among women currently using short-acting contraceptive methods.

**Methods:**

Institution-based cross-sectional study was conducted from March 19 to April 19, 2017. The study included 515 short-acting contraceptive users found in Gondar city who were selected from the study population using a systematic random sampling technique. A structured pre-tested questionnaire based on the theory of planned behavior was used to collect data. Both descriptive and analytical statistical procedures were used. Simple and multiple linear regression analyses were carried out. SPSS version 20 was used for the analysis of the data. Multiple linear regression was carried out to see the association between intention and sociodemographic variables, past contraception experience, attitude, subjective norm, and perceived behavioral control, and with 95% CI and a *p*-value of <0.05 was used to detect statistical significance.

**Results:**

The magnitude of intention to use long-acting contraceptive methods was 39.8%. Intention to use long-acting contraceptive methods was higher for women whose husbands were farmers when compared to women whose husbands were government employees (β =0.49, 95% CI: 0.41, 0.72). Number of children wanted (β = −0.19, 95% CI: −0.28, −0.1), attitude (β = 0.34, CI: 0.23, 0.46), subjective norm (β = 19, 95% CI: 0.12, 0.27) and perceived behavioral control (β = 0.18, 95% CI: 0.06, 0.3) were factors significantly associated with intention to use long-acting contraceptive methods.

**Conclusions:**

Intention to use long-acting contraceptive methods was low. Interventions that focus on women's attitudes toward long-acting contraceptive methods and support groups, peer education, social mobilization, and persuasive communication could promote the intention to use long-acting contraceptive methods.

## Background

Family planning can prevent at least 25% of all maternal deaths by allowing women to delay motherhood, prevent unintended pregnancies, and avoid unsafe abortions. It also protects women from sexually transmitted infections, including HIV/AIDS (1).

In many sub-Saharan African countries, long-acting contraceptive methods practice have the lowest rates and are sometimes a missing component of national family planning programs (2). For example, in Kenya, the contraceptive prevalence rate (CPR) increased from 17% in 1984 to 19% in 2003. Despite its increase, a decreasing proportion (from 31 to 2%) of intra-uterine device (IUD) use in the national method mix has been documented (3).

In Ethiopia, the contraceptive magnitude at a national level has quadrupled from 6% in 2000 to 27% in 2011 (4). The unmet need for family planning is 25 and 22% for Ethiopia and Amhara Regional State, respectively (1).

In most Sub-Saharan African countries like Malawi and Rwanda, diversifying the method mix is still a big challenge. Injectables predominate, accounting for 76% of modern method use, whereas long-acting contraceptive methods (LACMs) contribute only 4.2% and among these implants account for about 3.4% and intrauterine contraception devices (IUCD) only 0.3% (4).

Over the long term, LACMs are 20 times more effective than short-term contraceptives (5). If only one in five sub-Saharan African women now using pills or injectables were to switch to an implant (6), more than 1.8 million unintended pregnancies would be averted in 5 years, resulting in almost 600,000 fewer abortions and 10,000 fewer maternal deaths (7).

Currently, the Ministry of Health (MoH) has been giving due attention to the expansion of LCMS. To achieve this goal, MoH has practiced the task shift, which enabled the Health Extension Workers to provide implants at the community level, and the scale-up of intrauterine contraceptive devices has been initiated in more than 100 district hospitals and permanent contraceptive methods at selected health centers and hospitals (8, 9). However, the contraceptive prevalence rate is still highly dependent on short-term family planning methods with low utilization of LACMs and the unmet need for family planning is still high for spacing births (16%) and limiting (9%) (1).

Since the intention is the best predictor of behavior (utilization of LACMs), this study assessed the intention to use long-acting contraceptive methods and identified factors affecting it among short-acting contraceptive users using the theory of planned behavior. Results from this study will help in developing evidence-based LACMS health promotion programs.

## Methods

### Study design and period

The institution-based cross-sectional study was conducted from March to April 2017.

### Sample size and sampling procedure

The sample size was determined by the single population proportion formula using the EPI INFO stat calc program with the assumption of population size of 154,767, 95% level of confidence, 4% of marginal error, and taking the proportion of intention to use long-acting contraceptives, implants 71.3%, IUCD 24.0% (10). Considering a 5% non-response rate, the final sample size became 519. The study participants were selected by using a systematic random sampling technique and the sampling interval was 2.

### Data collection instruments and procedures

Data were collected using a pre-tested, structured questionnaire having two parts. In the first section, information about socio-demographic, economic, and reproductive history was collected. In the second section, the theoretical constructs were measured. The number of questions for each construct varied between 3 and 10. Forty-four items of Theory of Planned Behavior (TPB) constructs were assessed on a 5-point Likert scale, and they were scored into respective constructs for analysis. Since most women are not familiar with the phrase, long-acting contraceptive methods, it was defined in the questionnaire as long-acting contraceptive methods are methods that are used for three and/or more 3 years, IUCD and implant. The questionnaire was initially prepared in English and translated into Amharic (the official language fluently spoken by all participants) and again translated back to English to check for any inconsistencies or distortions in the meaning of words and concepts. The reliability of the tool was checked using Cronbach's alpha reliability test with a score of 0.82 (95% CI, 0.801–0.837).

### Data processing and analysis

All collected data were entered into Epi info version 7.0 and exported to SPSS version 20 statistical software for its analysis. Descriptive analysis was used to see the frequency distribution, mean, and SD. A correlation analysis was done between indirect and direct theory of planned behavior (TPB) variables to see the correlation between them. Multiple Linear regression analysis was computed to test the strength and direction of association between the dependent variable and independent variables. *R*^2^ was used to test the ability of explanatory variables to explain dependent variables. An unstandardized β coefficient was used to interpret the effect of predictors on the intention to use LACMs. The assumption of normality was checked statistically and it was normally distributed. A test of homoscedasticity using White's test was conducted. All the results supported the assumption of homoscedasticity. The linearity assumption was checked using a scatter plot of the standardized residuals vs. the predicted values from the regression analysis. Multicollinearity assumptions were tested by the variance inflation factor (VIF), and the value of all variables was below ten. Variables with a *p*-value of <0.05 at 95% CIs were considered statistically significant.

### Operational definitions

#### Intention

To use an IUCD or implant within 1 year.

#### Attitude

The women's overall evaluation of the benefits and risks of long-acting contraceptives.

#### Subjective norm

Women's assessment of the social pressure to use or not to use long-acting contraceptives and their motivation to comply.

#### Short-term contraceptives

Pills and Depo Provera injections that are taken daily and every 3 months, respectively.

## Results

### Demographic, and socioeconomic characteristics of the participants

A total of 515 women of short-acting contraceptive users participated in this study with a 99.2% response rate. The respondents' age ranged from 15 to 48 years with a mean age of 26.77 (SD ± 6.1) years. One hundred sixty-two (31.5%) participants were in the age category of 25–29. Of the study participants, 453 (88%) were married. The majority of the women 468 (90.9%) were Orthodox Christians and 478 (92.8%) women were Amhara by ethnicity.

Concerning educational status, 167 (32.4%) of the respondents were unable to read and write at all and 124 (27.4%) of their husbands were unable to read and write. Regarding the occupation of the respondents, 250 (48.5%) were housewives. The median monthly income of the respondents was $74 (Table 1).

**Table 1:** Socio-demographic and economic characteristics of short-acting contraceptive user women in Gondar city, Northwest Ethiopia, April 2017.

**Characteristic**		**Freq**.	**Percent**
Age category	15–19	83	16.1
	20–24	113	21.9
	25–29	162	31.5
	30–34	110	21.4
	35–49	47	9.1
Marital status	Married	453	88.0
	Single	62	12.0
Religion	Orthodox	468	90.9
	Muslim	35	6.8
	Protestant	12	2.3
Ethnicity	Amhara	478	92.8
	Tigray	19	3.7
	Others	18	3.5
Residence	Rural	122	23.7
	Urban	393	76.3
Education	can't read and write	167	32.4
	1–4th grade	45	8.7
	5–8th grade	95	18.4
	9–12th grade	135	26.2
	12+	73	14.2
Occupation	House wife	250	48.5
	Government employee	41	8.0
	Self employed	96	18.6
	Daily laborer	52	10.1
	Farmer	14	2.7
	Student	59	11.5
	Other	3	.6
Husband's education	can't read and write	124	27.4
	1–4th grade	41	9.1
	5–8th grade	72	15.9
	9–12th grade	82	18.1
	12+	134	29.6
	Total	453	100
Husband's occupation	Government employee	93	20.5
	Self employed	168	37.1
	Daily laborer	73	16.1
	Farmer	99	21.9
	Student	19	4.2
	Other	1	0.2
	Total	453	100
Income category	<750 birr	49	9.5
	750–1,500	105	20.4
	1,500–2,250	137	26.6
	2,250–3,000	100	22.3
	>3,000	58	11.3
	I don't know	66	12.8

### Intention to use long-acting contraceptive methods

The intention to use LACMs was 39.8%. The majority of the women (72.1%) intended to use an implant and 27.9% intended to use IUCD.

Attitude-wise, about 213 (41.4%) participants believed that using LACMs was unpleasant. About 174 (33.8%) of the participants opposed that using LACMs would help them work effectively. Nearly 181 (39.7%) of the participants replied that using LACMs would not help them to have a satisfying relationship with their husbands. About 213 (41.4%) of the women agreed using LACMs would subject them to different problems.

Concerning subjective norms, about 214 (41.6%) of the women answered that most people who are important to them think that they should not use LACMs. Two hundred twenty-three (43.3%) of the respondents disagreed that most people whose opinions they valued would approve of their using LACMs.

Concerning perceived behavioral control, about 262 (50.9%) of the respondents replied that it is difficult for them to use LACMs and 132 (29.7%) of them answered that it is easy for them to use them. Two hundred ninety-four (57.1%) of the women were confident that if they wanted to, they could use LACMs within 1 year. Nearly 360 (69.9%) of the women answered that it is possible to use LACMs (Table 2).

**Table 2:** Intention to use long-acting contraceptive methods among short-acting contraceptive user women in Gondar city, northwest Ethiopia, March 19 to April 19, 2017.

**Variables**		**Count**	***N* %**
**Subjective norm**			
Most people who are important to me think that I should use LACMs	Very unlikely	113	21.9
	Unlikely	214	41.6
	Uncertain	97	18.8
	Likely	80	15.5
	Very likely	11	2.1
**Attitude toward LACM**			
To use LACMs is	Very harmful	26	5.0
	Harmful	195	37.9
	Not sure	103	20.0
	Beneficiary	176	34.2
	Very beneficiary	15	2.9
To use LACMs is	Extremely worthless	5	1.0
	Worthless	73	14.2
	Not sure	85	16.5
	Valuable	331	64.3
	Extremely valuable	21	4.1
To use LACMs is	Extremely unpleasant	46	8.9
	Unpleasant	213	41.4
	Not sure	144	28.0
	Pleasant	104	20.2
	Extremely pleasant	8	1.6
**Perceived behavioral control**			
For me to use LACMs is	Extremely difficult	40	7.8
	Difficult	262	50.9
	Not sure	60	11.7
	Easy	137	26.6
	Extremely Easy	16	3.1
I am confident that if I wanted to I could use LACMs in 1 yr	Definitely false	16	3.1
	False	85	16.5
	Not sure	92	17.9
	True	294	57.1
	Definitely True	28	5.4
To use LACMs is	Extremely impossible	14	2.7
	Possible	73	14.2
	Not sure	48	9.3
	Possible	360	69.9
	Extremely possible	20	3.9
**Normative belief**			
Health professionals think I better use LACMs	Very unlikely	8	1.6
	Unlikely	24	4.7
	Uncertain	63	12.2
	Likely	243	47.2
	Very likely	177	34.4
My parents think that I should use LACMs	Very unlikely	99	19.2
	Unlikely	137	26.6
	Uncertain	191	37.1
	Likely	77	15.0
	Very likely	11	2.1
My close friends think that I should use LACMs	Very unlikely	68	13.2
	Unlikely	190	36.9
	Uncertain	143	27.8
	Likely	107	20.8
	Very likely	7	1.4
My relatives think I should use LACMs	Very unlikely	60	11.7
	Unlikely	171	33.2
	Uncertain	209	40.6
	Likely	70	13.6
	Very likely	5	1.0
–My husband thinks I should use LACMs	Very unlikely	138	30.3
	Unlikely	165	36.2
	Uncertain	54	11.8
	Likely	76	16.7
	Very likely	23	5.0

### Factors affecting intention to use long-acting contraceptive methods

The score on intention to use LACMs increased by 0.34 for every unit increase in the score of attitudes (β = 0.34, CI: 0.23, 0.46). For every unit increase in the score of subjective norms, the score of intention to use LACMs increased by 0.19 (β = 19, 95% CI: 0.12, 0.27). For every unit increase in the score of perceived behavioral control the score of intention to use LACMs increased by 0.18 (β = 0.18, 95% CI: 0.06, 0.3).

For women whose husbands were farmers, the score of intention to use LACMs increased by 0.43 when compared to women whose husbands were government employees (β = 0.49, 95% CI: 0.41, 0.72). For every unit increase in the number of children wanted in the future, the score of intention to use LACMs decreased by 0.19 (β = −0.19, 95% CI: −0.28, −0.1) (Table 3).

**Table 3:** Factors associated with intention to use LACMs among short-acting contraceptive user women in Gondar city, North West Ethiopia, March 19 to April 19, 2017.

**Variables**	**β**	**95% CI**
Constant	−2.31	(−3.03, −1.6)
**Occupation**
House wife	0	
Government employee	−0.061	**(**−0.42, 0.30**)**
Self-employed	0.109	(−0.15, 0.37)
Laborers	−0.033	(−0.37, 0.30)
Farmers	−0.255	(−0.74,0.23)
Student	0.477	(−0.04, 0.99)
Others	−0.050	(−1.2, 1.11)
**Husband occupation**
Government employee	0	
Self-employed	0.102	(−0.15, 0.35)
Laborer	0.098	(−0.22, 0.41)
Farmer	0.430^**^	(0.14, 0.72)
Student	−0.042	(−1.0, 0.93)
No of children wanted	−0.186^*^	(−0.28, −0.1)
Attitude	0.343^*^	(0.23, 0.46)
Subjective norm	0.194^*^	(0.12, 0.27)
Perceived behavioral control	0.183^**^	(0.06, 0.30)

** *p*-value < 0.005;

* *p*-value < 0.0001,

*R*^2^ = 40.1%. 0 = reference category.

## Discussion

This study aimed to assess the intention of using long-acting contraceptive methods and associated factors among women currently using short-acting contraceptive methods. The overall magnitude of intention to use LACMs within 1 year was 39.8% (95% CI: 35.7, 44.3). It was slightly lower than the study done in Adigrat which was 48.6% (11, 12). The difference could be due to disparities in the study settings and myths and misconceptions about long-acting contraceptive methods. This is supported by a study in which having fears that family planning would harm a woman's womb may lower a woman's intentions to use methods requiring procedures, such as the IUD (13–15). However, this result was in line with the study done in Wolaita which was 38% (16).

In this study attitude, subjective norm, and PBC were all statistically significant factors of intention to use long-acting contraceptive methods. These results support Ajzen's theoretical assumptions (17–19). The more women have a positive attitude, believe that people around them would approve of their actions, and believe that they have a high degree of control over using IUCD and implants, the greater will be their intention to use LACMs. This is in line with a study done in which a positive attitude was associated with more intention to use LACMs (20, 21).

Attitude plays the most important role in predicting women's intention to use LACMs. For each unit increase in the score of attitudes, the score of intention to use LACMs increased by 0.34. Also the study in Wolaita, Ethiopia documented that those women who had positive attitudes had a higher intention to use LACMs than women who had negative attitudes (15, 22, 23). Most of the women thought using these methods would subject them to a different problem, and they thought it would not allow them to effectively carry out their daily activities, hence in fear of the perceived side effects they did not intend to use LACMs.

Subjective norm denotes that women felt significantly the social pressure from others. According to the interviews, there was high pressure exerted mainly by spouses, relatives, and friends who did not favor the intention of using long-acting contraceptive methods. Even if a majority of the respondents perceived health professionals wanted them to use LACMs, they did not think relatives, husbands, and close friends would want them to use LACMs. This might be the reason why regardless of health professionals' efforts women failed to intend to use LACMs.

The current study also reported that direct perceived behavioral control is the predictor of intention to use LACMs. This is because most respondents agreed that if they wanted to use LACMs, they could use LCMS. This means that if they wanted to use LACMs, no barrier could stop them from using LACMs. However, a substantial number of respondents said LACMs were difficult for them. This might be due to fear of pain during insertion of implant or IUCD.

The number of children wanted was also significant (β = −0.19, 95% CI: −0.28, −0.1). The number of children who wanted to increase by a unit, and the score of intention to use LACMs decreased by 0.19. This finding was supported by the study done in Adigrat (11). If they had a plan to conceive soon, they preferred short-acting contraceptive methods. It might also be due to fear of side effects and misconceptions about LACMs, which could harm the womb and cause infertility after use of LACMs.

## Conclusions

The intention to use LACMs was low. Attitude, subjective norm, perceived behavioral control, the number of children wanted in the future, and husbands' occupations were factors significantly associated with using LACMs. Therefore, programs aimed at increasing LACMs utilization need to address these identified factors of LACMS against users of short-acting contraceptive methods.

### Limitation of the study

Social desirability bias was the limitation of the study because respondents perceived intending to use LACMs is expected of them. Therefore, there is some potential for reporting bias, which may have occurred because of the respondent's interpretation of the questions or desire to report their emotions in a certain way or simply because of inaccuracies in responses.

## Data availability statement

The raw data supporting the conclusions of this article will be made available by the authors, without undue reservation.

## Ethics statement

The studies involving human participants were reviewed and approved by Ethical clearance was obtained from Ethical Review Committee of Institute of Public Health, University of Gondar. All methods were performed in accordance with the relevant guidelines and regulations. Permission to conduct the research was obtained from Gondar city administration. Written Informed consent was obtained from respondents who were selected to participate in the study after explaining the purpose of the study and consent was obtained from the parents of minor study participants. Written informed consent for participation was not required for this study in accordance with the national legislation and the institutional requirements.

## Author contributions

EF, TA, RB, and AN conceived, designed the idea, and analyzed the data. MW and ZA wrote and critically reviewed the manuscript. All authors have read and approved the final draft of the manuscript.

## Conflict of interest

The authors declare that the research was conducted in the absence of any commercial or financial relationships that could be construed as a potential conflict of interest.

## Publisher's note

All claims expressed in this article are solely those of the authors and do not necessarily represent those of their affiliated organizations, or those of the publisher, the editors and the reviewers. Any product that may be evaluated in this article, or claim that may be made by its manufacturer, is not guaranteed or endorsed by the publisher.

## Abbreviations

BI: behavioral intention
CPR: contraceptive prevalence rate
EDHS: ethiopian demographic health survey
FP: family planning
HC: health center
IUD: intra uterine device
LACMs: long-acting contraceptive methods
MoH: ministry of health
PBC: perceived behavioral control
RH: reproductive health
SPSS: statistical package for social sciences
SSA: sub- saharan Africa
TPB: theory of planned behavior
WHO: world health organization.
